# Ocular Surface Disease Signs and Symptoms in Patients with Pseudoexfoliative Glaucoma: A Case—Control Study

**DOI:** 10.3390/vision6010011

**Published:** 2022-02-08

**Authors:** Maria Dermenoudi, Artemis Matsou, Christina Keskini, Eleftherios Anastasopoulos

**Affiliations:** 1 Health Center of Neapolis, 56727 Thessaloniki, Greece; maraki56224@hotmail.com; 2 Ophthalmology Department, Queen Victoria Hospital NHS Foundation Trust, East Grinstead RH19 3DZ, UK; art.matsou@gmail.com; 3 1st Department of Ophthalmology, Aristotle University of Thessaloniki, 54621 Thessaloniki, Greece; christinakeskini@gmail.com; 4 2nd Department of Ophthalmology, Aristotle University of Thessaloniki, 56403 Thessaloniki, Greece

**Keywords:** pseudoexfoliation, pseudoexfoliative glaucoma, ocular surface disease, primary open angle glaucoma, dry eye syndrome

## Abstract

Purpose: The present study evaluates the differences in the prevalence of the signs and symptoms of ocular surface disease (OSD) in patients with PEX glaucoma (PEXG), compared to other glaucoma types (non-PEXG). Methods: Patients with non-PEXG and PEXG were prospectively examined for the presence and severity of OSD signs and questioned for symptoms using the OSDI (ocular surface disease index) questionnaire. Results: 116 patients were prospectively enrolled (58 non-PEXG and 58 PEXG). PEXG subjects who were older, had lower central corneal thickness (CCT) values, at a more advanced glaucoma stage and required more IOP lowering drops. OSD signs were prevalent in both groups: conjunctival hyperemia (74.5% non-PEXG vs. 94.8% PEXG), eyelid redness (70.7% vs. 96.6%), conjunctival (74.1% vs. 93.1%) and corneal fluorescein staining (81% vs. 93.1%) and abnormal TFBUT (82.8% vs. 87.9%). When adjusted for potential confounders, (older age, thinner CCT, more advanced glaucoma in PEXG) eyelid redness remained the only parameter significantly associated with PEXG, being 11 times more likely to occur in this group (*p* = 0.037). Conclusion: Subjects with PEXG presented a higher frequency of signs of OSD compared to other glaucoma types. When accounting for confounding factors, the only difference between the groups was the significantly higher presence (11 times more likely) of eyelid redness in PEXG, suggesting, in addition to glaucoma treatment, the impact of PEX on ocular surface integrity.

## 1. Introduction

Pseudoexfoliation (PEX) syndrome is considered the most identifiable cause of secondary open angle glaucoma (pseudoexfoliative glaucoma, PEXG) [1], characterized by the gradual accumulation and deposition of a whitish fibrillar substance of unknown origin, most notably in intraocular anterior segment structures, such as the anterior lens capsule, iris and pupillary margin, iridocorneal angle, zonules, ciliary body and corneal endothelium [2]. Extraocular tissues, such as conjunctival goblet cells and accessory lacrimal glands, also appear to be affected by PEX deposition, as demonstrated in conjunctival biopsies and impression cytology studies [3,4,5,6]. The abnormal or reduced function of such structures may lead to alterations of the mucous layer of the tear film, resulting in dry eye syndrome (DES) and ocular surface disease (OSD) [7], implying a direct impact of PEX on tear film stability. These findings are further supported by the fluorescein angiography of the conjunctiva in PEX subjects, exhibiting the damage of the regular limbal vascular pattern with anterior ciliary vessel congestion and patches of neovascularization [8]. Therefore, patients with pseudoexfoliative material may allegedly suffer from more impaired tear secretion and ocular surface disease. 

The chronic use of topical anti-glaucoma medications in glaucoma patients has been thoroughly studied and demonstrated to alter ocular surface and tear film function [9,10,11]. Preservatives contained in glaucoma eye drops, with benzalkonium chloride (BAK) being the most widely used, have long been accused of provoking or aggravating OSD, mainly through corneal epithelial toxicity, conjunctival squamous metaplasia, loss of goblet cells, ocular surface inflammation and tear film disruption [12]. 

The combination of the iatrogenic-induced OSD in glaucoma patients under topical treatment and the suspected inherent susceptibility of individuals with pseudoexfoliative material to OSD pose a particular concern for PEXG patients for the development of severe DES and OSD. The implications on quality of life and patient adherence to treatment for PEXG subjects could be substantial.

The purpose of this prospective case series is to evaluate the prevalence of the signs and symptoms of ocular surface disease in patients receiving intraocular pressure (IOP) lowering medication for PEX (PEXG) and non-PEX glaucoma, and to compare the severity of OSD between these groups. In addition, we aim to investigate the impact of confounding factors, such as the treatment duration and intensity, on OSD severity in this cohort. 

## 2. Patients and Methods

This prospective observational cross-sectional study was conducted in the Department of Ophthalmology of Papageorgiou General Hospital, Thessaloniki, Greece. PEX glaucoma (PEXG) patients and non-PEXG subjects presenting in the glaucoma outpatient clinic and receiving topical treatment with IOP-reducing agents for a minimum of 6 months were prospectively enrolled over an 8-month period (August 2017–April 2018). Written informed consent was obtained from all participants. The study was approved by the local Ethical Research Committee and was conducted in accordance with the principles of the Declaration of Helsinki.

Patients with clinically detectable pseudoexfoliation material on the lens capsule, iris and pupillary border and corneal endothelium in at least one eye, along with evidence of glaucomatous optic neuropathy, receiving IOP-lowering drugs, were included in the PEXG group. Individuals under topical therapy for other types of glaucoma (primary open angle, pigmentary and chronic angle-closure glaucoma) or high-risk ocular hypertension (OH) with no evidence of pseudoexfoliation deposits comprised the non-PEXG group. Subjects presenting with other recognizable causes of impaired tear film production or integrity were excluded from our cohort. The exclusion criteria included prior intraocular or corneal surgery; the wearing of contact lenses; systemic disease (thyroid eye disease, Sjogren’s syndrome, sarcoidosis, rheumatoid arthritis and any autoimmune condition associated with dry eye syndrome); corneal pathology (corneal dystrophies, ectasias and corneal scarring); eyelid abnormalities (entropion, ectropion and lagophthalmos); pterygium; nasolacrimal drainage obstruction or occlusion of the lacrimal puncta; history of allergic/vernal conjunctivitis; systemic medications linked to dry eye, such as antidepressants, and other significant ophthalmic pathologies. 

All participants underwent a detailed standard ophthalmic assessment as routinely performed in a single visit at the outpatient glaucoma clinic, including best-corrected visual acuity (BCVA) using the Snellen chart, IOP measurement by Goldmann applanation tonometry, gonioscopy with a Volk gonio lens, slit lamp biomicroscopy and dilated fundus examination. Medical notes were carefully reviewed to ensure eligibility for recruitment. The basic demographic data included age, gender, type of glaucoma, duration of glaucoma diagnosis and duration of treatment with eye drops. Further parameters documented for all participants were the total number of daily glaucoma eye drops, number of preserved glaucoma eyedrops, central corneal thickness (CCT) measurement (pachymetry) and mean deviation (MD) of the most recent visual field (Zeiss Humphrey Visual Field Analyser, Zeiss, Jena, Germany). Patients were also examined for signs of ocular surface disease by two separate investigators (MD, AM), with a consensus on OSD grading being reached in case of disparity by the principal investigator (EA). One eye per patient was selected for enrolment in the study. In case of bilaterally applied topical IOP-lowering treatment, the right eye was included in the analysis. In cases of unilateral glaucoma treatment, the eye being treated was selected. 

The signs of ocular surface disease selected for clinical evaluation were the severity of eyelid redness, conjunctival hyperaemia, fluorescein conjunctival and corneal staining and tear film break-up time (TFBUT). We used the Oxford grading scale to assess corneal and conjunctival staining (absent, mild, moderate, and severe) [13] and the Efron grading scale to assess the presence and intensity of conjunctival and eyelid redness [14], comparing against illustrated images produced to standardize anterior eye evaluations. Tear film break-up time was measured before the instillation of any eye drops or manipulation of the eyelids. We used fluorescein strips without wetting in order to avoid the unwanted effect of the wetting fluid on tear film stability and placed them in the lower fornix. The patient was then asked to blink several times and examined under cobalt blue light with a broad beam. TFBUT was subsequently recorded in seconds passed before the appearance of dark dry areas (<5 s, 5–10 s, and >10 s), with values < 10 s defined as abnormal. Environmental conditions, including room temperature, humidity and ventilation, were similar for all patients. 

In order to evaluate the subjective severity of dry eye symptoms, we provided our patients with the ocular surface disease index (OSDI) questionnaire at the beginning of the consultation. OSDI questionnaire has been reported as a validated and reliable tool to assess the impact of OSD on quality of life [15]. Developed by Allergan Inc. to discriminate between mild, moderate and severe eye disease, the questionnaire consists of 12 questions, divided into 3 different subgroups: ocular discomfort (5 items), ocular symptoms during daily activities (4 items), and ocular symptoms caused by environmental triggers (3 items) within the previous 4 weeks. Each item is scored from 0 to 4, where 0 indicates none of the time; 1, some of the time; 2, half of the time; 3, most of the time; and 4, all the time. The total OSDI score was then calculated for each patient from 0 to 100 based on the OSDI formula [(sum of scores) × 25]/No of questions answered, with higher scores representing greater disability. Normal OSDI was denoted for scores <12, mild OSDI for scores 13–22, moderate for 23–32, and severe for >33, as previously reported [16].

## 3. Statistical Analysis

Statistical analyses were performed using the Statistical Package for the Social Sciences ver. 23.0 (IBM Corp., Armonk, NY, USA). Mean value and standard deviation were used to describe normally distributed continuous variables, while median and range [min, max] for non-normally distributed continuous variables. Percentages were used to describe categorical variables. Subjects with pseudoexfoliative glaucoma were compared to subjects with other glaucoma types with respect to all continuous and categorical variables. *T*-test and Mann–Whitney *U* test were used for comparisons regarding normally distributed and non-normally distributed continuous variables, respectively. Chi-square test was used for comparisons with categorical variables and Fisher’s exact test when at least one cell had expected count less than 5. 

Logistic regression was used to investigate the association between the presence of ocular surface disease signs and the presence of pseudoexfoliative glaucoma. Binary univariate logistic regression was conducted to estimate crude odds ratio (OR), whilst logistic regression model was also performed in order to adjust for potential confounders. Odds ratios (OR) and 95% confidence intervals (95% CI) are presented. A value of *p* < 0.05 was considered statistically significant.

## 4. Results

One-hundred and sixteen (116) eyes of one-hundred and sixteen patients meeting the inclusion criteria were prospectively enrolled. Pseudoexfoliative glaucoma was identified in 58 (50%) subjects, while 58 had other types of glaucoma; of the latter group, the majority presented with POAG (*n* = 40), 8 with OH, and the remaining with normal-tension (NTG), pigmentary (PG) and chronic closed-angle glaucoma (CCAG) (total non-PEXG = 58). The distribution of participants’ basic characteristics is detailed in Table 1.

The groups differed significantly in age distribution, with the mean age in the PEXG group being 75.22 ± 6.38 and in non-PEXG 66.71 ± 11.45, (*p* < 0.001), as well as in central corneal thickness (CCT) with PEXG subjects having significantly thinner cornea (525.40 (±36.61) vs. 546.89 (±34.81), (*p* = 0.004), as shown in Table 1. There were no statistical differences with regard to gender and duration of treatment. The mean deviation on the most recent visual field was worse in PEXG subjects [−6.32 dB (−32.67, 1.00)] compared to non-PEXG [−2.84 dB (−17.40, 1.83)] (*p* = 0.001). The total number of drops and number of preserved drops used per day were also different between groups, with PEXG subjects requiring more daily eyedrops to achieve IOP control (total number of drops (median) 2 (1–5), number of preserved drops (median) 2 (0–5)) compared to non-PEXG (total number of drops (median) 1 (1–3), number of preserved drops (median) 1 (0–3)) (*p* < 0001). The mean OSDI score was comparable, although PEXG subjects reported overall worse symptoms (OSDI score (median) 22.61 (0–84.3)) compared to non-PEXG (OSDI score (median) 19.05 (0–94.4)) (*p* = 0.137).

A significant number of patients in both groups exhibited clinical signs of ocular surface disease: conjunctival hyperaemia 89.7%, eyelid redness 83.6%, conjunctival fluorescein staining 83.6%, corneal fluorescein staining 87.1%, and abnormal TFBUT (<10 s) 85.4%. The mean values ± SD and median values [min, max] for all OSD parameters studied are shown in Table 1. In the univariate logistic regression analysis, eyelid redness and conjunctival fluorescein staining were significantly worse in subjects of the PEXG group (OR = 11.61; CI (2.54, 53.05), *p* = 0.002 and OR = 4.70; CI (1.45, 15.22), *p* = 0.01, respectively) compared to non-PEXG patients. Although there was a trend towards a positive association between PEXG and corneal staining as well as conjunctival hyperaemia, neither of these reached the level of statistical significance (OR = 3.16; CI (0.94, 10.58), *p* = 0.062 and OR = 3.36; CI (0.86, 13.14), *p* = 0.081, respectively). TFBUT was not significantly associated with the presence of PEXG (*p* = 0.433).

After stepwise adjusting for parameters, which could impact the outcome (age, gender, CCT, glaucoma duration, visual field MD, number of preserved and total number of drops), the only parameter identified to differ significantly between the groups was the presence of eyelid redness, with PEXG subjects being 10.77 times more likely to present with eyelid redness compared to non-PEXG cases. The outcomes of the multivariate regression model are displayed in Table 2. 

## 5. Discussion

In our study, both OSD signs and symptoms were highly prevalent in both groups. Eyelid redness and conjunctival fluorescein staining were statistically more frequent in PEXG compared to non-PEXG eyes. After controlling for several confounders, PEXG eyes, compared to those with non-PEXG, were 11 times more likely to present either moderate or severe eyelid redness. 

Ocular surface disease is a substantial concern in glaucoma patients and is thought to be provoked or aggravated by the chronic use of IOP-lowering agents, with the preserved ones being the principal culprits. Previous studies have highlighted the higher prevalence of OSD symptoms in glaucoma patients under topical treatment [17,18], so much so that OSD can be considered a comorbidity of glaucoma. Glaucoma treatment side effects account for 10–15% of poor compliance, which in turn is responsible for approximately 10% of glaucomatous visual loss [19], underscoring the need to address the combined impact of glaucoma and OSD on quality of life and compliance to therapy [20]. A number of studies have looked into the association of glaucoma with OSD; however, data regarding the potential effect of specific glaucoma types on ocular surface integrity are scarce. In this study, we aimed to investigate whether pseudoexfoliative glaucoma poses an independent additional hazard for ocular surface impairment compared to other types of glaucoma. The deposition of pseudoexfoliative material in goblet cells and accessory lacrimal glands has been demonstrated in previous reports [4,5,6,8], and therefore may negatively affect tear secretion and tear film stability, further compromising the glaucoma-related OSD.

The participants of our study had similar baseline characteristics, apart from age and CCT, with PEXG patients being older (75.22 vs. 66.71) and with lower CCT measurements (525.40 μm vs. 546.89 μm). PEX is by definition an age-related systemic disorder, with PEX accumulation increasing with ageing [21,22], accounting for the age disparity in our cohort. The difference detected in CCT measurements in our study, with pseudoexfoliative eyes having significantly thinner CCT values than non-PEXG subjects, has also been described in the literature, although there are contrasting reports. Several authors advocate that PEX and PEXG eyes demonstrate thinner CCT compared to normal controls, eyes with OH and other glaucoma types [23,24,25,26,27], while other large-population-based studies report no significant differences [28,29,30,31] in CCT measurements between PEXG and control eyes. Although the association between PEX and CCT remains ambiguous, our results are in keeping with the decrease in corneal stromal cell density observed in PEX eyes [32]. Our PEXG cohort required more daily eye drops to achieve optimal IOP control compared to non-PEXG subjects, denoting the typically more aggressive course of PEXG, with high IOP at onset, faster rates of progression and poor response to medical therapy, especially when compared to primary open angle glaucoma [33]. Mean deviation values on visual fields testing were also worse in PEXG patients, revealing a more pronounced field loss and advanced stage of glaucoma in this group [34].

The connection between glaucoma and OSD signs [35] has been thoroughly explored in the literature, with a number of studies investigating the impact of glaucoma-related OSD on quality of life and the correlation of clinical presence and severity of OSD with patient-reported impact on quality of life [18,36]. However, the effect of pseudoexfoliation on tear film functions and subsequently on quality of life remains scantily investigated. In our study, we used the ocular surface disease index (OSDI) questionnaire, which has been validated for assessing the symptoms related to OSD and the impact of OSD on vision-related quality of life [15]. Median OSDI scores mostly fell into the “mild” or “moderate” category, with no significant difference between groups. However, the PEXG group tended to have worse OSDI scores, without reaching statistical significance. The majority of patients in both groups had abnormal OSDI scores along with clinical signs of OSD, signifying a meaningful correlation between signs and symptoms. Previous studies have found high correlations between OSDI scores and the presence and severity of clinical signs [15,37,38].

Ocular surface disease signs were universally present in the majority of our participants, clearly establishing the effects and burden of glaucoma treatment on ocular surface. When comparing the groups, the only OSD parameter that differed significantly after adjusting for confounding factors (age, gender, glaucoma duration, number of preserved drops and CCT) was eyelid redness, with PEXG subjects having significantly worse eyelid redness compared to non-PEXG individuals (10.77 times more likely). Conjunctival and corneal damage seemed to not differ after adjusting for these factors, and neither did TFBUT. This difference in the eyelid redness, its pathophysiology and the possible related clinical implications need to be further explored in additional studies. Eyelid redness is one of the markers of ocular surface inflammation, usually accompanied by symptoms of irritation, itching, scaling, crusting, eyelash misdirection or loss and peri-ocular dermatitis along with cosmetic concerns, especially for younger patients. It is a common reason for attendance to ocular surface clinics for patients treated with glaucoma preparations, often requiring the use of tear supplements and eyelid foams, increasing the number of medication applied daily. It also constitutes a reason for treatment modifications despite acceptable IOP control, or for the early employment of laser, surgical or minimally invasive surgical strategies. In our study, PEXG subjects reported overall worse symptoms (OSDI score (median) 22.61 (0–84.3)) compared to non-PEXG patients (OSDI score (median) 19.05 (0–94.4)) (*p* = 0.137), which should be taken into consideration despite the lack of a statistically significant difference.

To the best of our knowledge, this is the first prospective study of the evaluation of OSD signs and symptoms in pseudoexfoliative glaucoma compared to other glaucoma types, while accounting for confounders. However, there are some limitations in our study. Although we carefully recorded current treatment regimens for all patients with an emphasis on preserved IOP-lowering eye drops, it is fair to say that other IOP lowering agents used before enrolment may have contributed to dry eye syndrome and OSD. The calculation of cumulative preservative use was beyond the scope of our study, but can be considered in future studies to record the cumulative effect of preserved therapy more accurately. We did not use a Schirmer test, which is another useful screening tool for basal and reflex tear secretion. As demonstrated by the lack of significant difference in TFBUT between the groups (*p* = 0.433), it is possible that Schirmer values would also have been similarly low in both groups; however, it might be a valuable additional information in further studies. Abnormal Schirmer and TFBUT results in PEX and PEXG individuals compared to healthy controls have been described in previous research papers [3,6,39].

## 6. Conclusions

This is a prospective study comparing ocular surface signs and symptoms between patients with pseudoexfoliative and other types of glaucoma. Based on our results, there is a link between pseudoexfoliation and OSD. Possible factors leading to this could be the older age of these patients, the thinner CCT, the more aggressive progression of the disease and the more advanced glaucoma stage requiring a higher number of glaucoma drugs (preserved and non-preserved). When accounting for these confounding factors, eyelid redness remained the only clinical sign of OSD highly associated with the presence of PEXG. This may suggest an additional to glaucoma treatment risk for OSD in subjects with PEXG. PEXG patients, being more prone to debilitating OSD, should be treated carefully, summoning all existing strategies to alleviate the burden of medical management.

## Figures and Tables

**Table 1:** Characteristics and ocular surface disease signs in subjects with pseudoexfoliative glaucoma (PEXG) and in Subjects with non-PEXG (*n* = 116).

	All Subjects (*n* = 116)	PEXG (*n* = 58)	Non-PEXG (*n* = 58)	*p* Value *
Age (years)	73.00 [42.00, 92.00]	75.22 (±6.38)	66.71 (±11.45)	**0.000**
Gender (male)	44.00% (51/116)	48.30% (28/58)	39.70% (23/58)	0.350
Glaucoma Duration (months)	62.00 [5, 314.00]	62.00 [5.00, 266.00]	62.00 [8.00, 314.00]	0.586
Number of Drops	1.00 [1.00, 5.00]	2.00 [1.00, 5,00]	1.00 [1.00, 3.00]	**0.002**
Number of Preserved Drops	1.00 [0.00, 5.00]	2.00 [0.00, 5.00]	1.00 [0.00, 3.00]	**0.000**
Ocular Surface Disease Index (OSDI)	20.60 [0.00, 94.40]	22.61 [0.00, 84.30]	19.05 [0.00, 94.40]	0.137
OSDI (Grades)				0.543
Normal (<12)	33.60% (39/116)	29.30% (17/58)	37.90% (22/58)	
Mild (13–22)	23.30% (27/116)	22.40% (13/58)	24.10% (14/58)	
Moderate (23–32)	12.90% (15/116)	12.10% (7/58)	13.80% (8/58)	
Severe (>33)	30.20% (35/116)	36.20% (21/58)	24.10% (14/58)	
MD (dB) **	−4.39 [−32.67, 1.83]	−6.32 [−32.67, 1.00]	−2.84 [−17.40, 1.83]	**0.001**
Central Corneal Thickness (CCT) (μm) ***	535.81 (±37.17)	525.40 (±36.61)	546.89 (±34.81)	**0.004**
Conjuctival Hyperemia				0.179
No	10.30% (12/116)	5.20% (3/58)	15.50% (9/58)	
Mild	40.50% (47/116)	48.30% (28/58)	32.80% (19/58)	
Moderate	22.40% (26/116)	20.70% (12/58)	24.10% (14/58)	
Severe	26.70% (31/116)	25.90% (15/58)	27.60% (16/58)	
Eyelid Redness				**0.003**
No	16.40% (19/116)	3.40% (2/58)	29.30% (17/58)	
Mild	40.50% (47/116)	50.00% (29/58)	31.00% (18/58)	
Moderate	23.3% (27/116)	24.10% (14/58)	22.40% (13/58)	
Severe	19.80% (23/116)	22.40% (13/58)	17.20% (10/58)	
Corneal Fluorescein Staining				0.065
No	12.90% (15/116)	6.90% (4/58)	19.00% (11/58)	
Mild	37.90% (44/116)	32.80% (19/58)	43.10% (25/58)	
Moderate	24.10% (28/116)	31.00% (18/58)	17.20% (10/58)	
Severe	25.00% (29/116)	29.30% (17/58)	20.70% (12/58)	
Conjuctival Fluorescein Staining				**0.003**
No	16.40% (19/116)	6.90% (4/58)	25.90% (15/58)	
Mild	46.60% (54/116)	41.40% (24/58)	51.70% (30/58)	
Moderate	24.10% (28/116)	32.80% (19/58)	15.50% (9/58)	
Severe	12.90% (15/116)	19.00% (11/58)	6.90% (4/58)	
Break Up Time (BUT) (s)				0.668
>10	14.60% (17/116)	12.10% (7/58)	17.20% (10/58)	
5–10	30.20% (35/116)	29.30% (17/58)	31.00% (18/58)	
<5	55.20% (64/116)	58.60% (34/58)	51.70% (30/58)	

Data presented are in mean values (±SD), median values [min, max] or frequency % (number of subjects), as appropriate for each variable. * *p* value for difference in characteristics and ocular surface disease signs based on *t*-test, Mann–Whitney U test, chi-square test or Fisher’s exact test, as appropriate. ** After excluding patients with missing MD values: *n* = 104. *** After excluding patients with missing CCT values: *n* = 97. Bold stands for statistical significance.

**Table 2:** Risk of ocular surface disease signs and symptoms in subjects with pseudoexfoliative glaucoma (PEXG) compared to non-PEXG subjects.

	PEXG vs. No-PEXG Unadjusted Odds Ratio (OR) (95% CI) (*n* = 116)	*p* Value	Adjusted Odds Ratio (OR) (95% CI) * (*n* = 116)	*p* Value	Adjusted Odds Ratio (OR) (95% CI) ** (*n* = 97)	*p* Value	Adjusted Odds Ratio (OR) (95% CI) *** (*n* = 97)	*p* Value	Adjusted Odds Ratio (OR) (95% CI) ± (*n* = 97)	*p* Value	Adjusted Odds Ratio (OR) (95% CI) # (*n* = 97)	*p* Value
Conjunctival Hyperemia	3.36 (0.86, 13.14)	0.081	3.11 (0.66, 14.59)	0.149	5.43 (0.64, 45.69)	0.119	5.14 (0.62, 45.52)	0.128	3.63 (0.42, 30.95)	0.238	4.60 (0.46, 45.55)	0.191
Eyelid Redness	11.61 (2.54, 53.05)	**0.002**	10.65 (2.03, 55.74)	**0.005**	13.17 (1.48, 116.80)	**0.021**	13.12 (1.47, 117.19)	0.021	11.13 (1.22, 101.02)	**0.032**	10.77 (1.16, 99.99)	**0.037**
Corneal Fluorescein Staining	3.16 (0.94, 10.58)	0.062	2.52 (0.61, 10.37)	0.198	1.78 (0.41, 7.70)	0.437	1.77 (0.41, 7.67)	0.441	1.32 (0.30, 5.78)	0.706	1.17 (0.26, 5.19)	0.837
Conjunctival Fluorescein Staining	4.70 (1.45, 15.22)	**0.010**	3.68 (0.87, 15.56)	0.076	2.54 (0.53, 12.09)	0.240	2.49 (0.51, 12.13)	0.256	1.99 (0.37, 10.65)	0.417	1.81 (0.33, 9.81)	0.487
Break Up Time (BUT) < 10	1.51 (0.53, 4.30)	0.433	0.87 (0.23, 3.24)	0.839	0.48 (0.11, 2.15)	0.343	0.49 (0.11, 2.16)	0.347	0.39 (0.08, 1.7)	0.227	0.34 (0.07, 1.61)	0.179
OSDI Grade mild/moderate/severe	1.52 (0.72, 3.19)	0.262	1. 03 (0.42, 2.51)	0.932	0.762 (0.27, 2.14)	0.607	0.73 (0.25, 2.10)	0.569	0.57 (0.18, 1.79)	0.338	0.54 (0.16, 1.74)	0.302

* Adjusted for age, gender and number of drops. ** Adjusted for age, gender, number of drops and CCT. *** Adjusted for age, gender, number of drops, CCT and glaucoma duration. ± Adjusted for age, gender, number of preserved drops, CCT, glaucoma duration and MD (mean deviation). # Adjusted for age, gender, total number of drops, number of preserved drops, CCT, glaucoma duration and MD. Bold stands for statistical significance.

## Data Availability

Not applicable.
